# Protective effect of kaempferol glucoside against lipopolysaccharide-caused acute lung injury via targeting Nrf2/NF-κB/NLRP3/GSDMD: Integrating experimental and computational studies

**DOI:** 10.1016/j.jsps.2024.102073

**Published:** 2024-04-18

**Authors:** Wesam H. Abdulaal, Ulfat M. Omar, Mustafa Zeyadi, Dina S. El-Agamy, Nabil A. Alhakamy, Naif A. R. Almalki, Hani Z. Asfour, Mohammed W. Al-Rabia, Abdulrahim A. Alzain, Gamal A. Mohamed, Sabrin R.M. Ibrahim

**Affiliations:** a Department of Biochemistry, Faculty of Science, Cancer and Mutagenesis Unit, King Fahd Medical Research Center, King Abdulaziz University, Jeddah 21589, Saudi Arabia; b Center of Excellence for Drug Research and Pharmaceutical Industries, King Abdulaziz University, Jeddah 21589, Saudi Arabia; c Department of Biochemistry, Faculty of Sciences, King Abdulaziz University, Jeddah 21589, Saudi Arabia; d Princess Dr. Najla Bint Saud Al-Saud Center for Excellence Research in Biotechnology, King Abdulaziz University, Jeddah, 21589, Saudi Arabia; e Department of Pharmacology and Toxicology, Faculty of Pharmacy, Mansoura University, Mansoura 35516, Egypt; f Department of Pharmaceutics, Faculty of Pharmacy, King Abdulaziz University, Jeddah 21589, Saudi Arabia; g Mohamed Saeed Tamer Chair for Pharmaceutical Industries, King Abdulaziz University, Jeddah 21589, Saudi Arabia; h Experimental Biochemistry Unit, King Fahad Medical Research Centre, King Abdulaziz University, Jeddah, Saudi Arabia; i Department of Clinical Microbiology and Immunology, Faculty of Medicine, King Abdulaziz University, Jeddah 21589, Saudi Arabia; j Department of Pharmaceutical Chemistry, Faculty of Pharmacy, University of Gezira, Wad Madani 21111, Sudan; k Department of Natural Products and Alternative Medicine, Faculty of Pharmacy, King Abdulaziz University, Jeddah 21589, Saudi Arabia; l Preparatory Year Program, Department of Chemistry, Batterjee Medical College, Jeddah 21442, Saudi Arabia; m Department of Pharmacognosy, Faculty of Pharmacy, Assiut University, Assiut 71526, Egypt

**Keywords:** Acute lung injury, Computational studies, Health and wellbeing, Industrial development, Kaempferol 3-sophoroside-7-glucoside, Life on land

## Abstract

The current study explored the protective potential of kaempferol 3-sophoroside-7-glucoside (KSG) against acute lung injury (ALI). Pre-treatment with KSG effectively secured mice from ALI and showed similar efficaciousness to dexamethasone. KSG markedly increased the survival rate and alleviated lung pathological lesions induced by lipopolysaccharide (LPS). Furthermore, KSG attenuated differential and total cell counts in BALF (bronchoalveolar lavage fluid) and MPO (myeloperoxidase) activity. KSG counteracted the NF-κB (nuclear factor-κB) activation and significantly ameliorated the downstream inflammatory cytokine, TNF-α (tumor necrosis factor-α). Simultaneously, KSG suppressed the over-expression of NLRP3 (NOD-like receptor protein 3**)**, caspase-1, and pro-inflammatory cytokine interleukin IL-1β (interleukine-1β) and prohibited the elevation of the pyroptotic parameter GSDMD-N (N-terminal domain of gasdermin D) induced by LPS challenge. In addition, KSG significantly enhanced Nrf2 (nuclear-factor erythroid-2-related factor) and HO-1 (heme-oxygenase-1) expression. Meanwhile, KSG mitigated lipid peroxidative markers (malondialdehyde, protein carbonyl and 4-hydroxynonenal) and boosted endogenous antioxidants (superoxide dismutase/reduced glutathione/catalase) in lung tissue. *In silico* analyses revealed that KSG disrupts Keap1-Nrf2 protein–protein interactions by binding to the KEAP1 domain, consequently activating Nrf2. Specifically, molecular docking demonstrated superior binding affinity of KSG to KEAP1 compared to the reference inhibitor, with docking scores of −9.576 and −6.633 Kcal/mol, respectively. Additionally, the MM-GBSA binding free energy of KSG (−67.25 Kcal/mol) surpassed that of the reference inhibitor (−56.36 Kcal/mol). Furthermore, MD simulation analysis revealed that the KSG-KEAP1 complex exhibits substantial and stable binding interactions with various amino acids over a duration of 100 ns. These findings showed the protective anti-inflammatory and anti-oxidative modulatory efficiencies of KSG that effectively counteracted LPS-induced ALI and encouraged future research and clinical applications of KSG as a protective strategy for ALI.

## 1 Introduction

ALI/ARDS (acute lung injury/acute respiratory distress syndrome) are critical morbid clinical conditions that are mainly manifested by sever inflammatory response of the lung (Ahmed et al., 2018, Abdallah et al., 2020). Many factors have been identified to cause ALI/ARDS as viral infection, drugs, high-pressure ventilation, and other systemic diseases. Till now, no effective treatment exists, and patients can only receive supportive treatment. So, searching for new therapeutics for treatment of ALI/ARDS is necessary. ALI/ARDS pathogenesis has not been totally established. Severe inflammation, oxidative stress and apoptosis are the major players in the pathogenic pathway of ALI/ARDS (Ammar et al., 2013, Wu et al., 2021). NLRP3 (NOD-like receptor protein-3) and NF-κB (nuclear-factor kappa-B) inflammasome are cornerstones in the development of ALI/ARDS. In normal conditions, NF-κB exists in an inactive state in the cytoplasm as it is captured with IκB (IkappaB kinase). Stimuli as pro-inflammatory factors and ROS (reactive oxygen species) boost the IκB phosphorylation and the NF-κB release which enters the nucleus and stimulates the expression of inflammatory mediators as ILs (interleukins) and TNF-α (tumor necrosis factor-alpha). Agents that hinder the NF-κB signaling activation were documented to confer protection against ALI (Tang et al., 2021, Cai et al., 2022). The role of NLRP3 in inciting the inflammation during ALI is not less important than NF-κB. NLRP3 inflammasome is a complex cytosolic receptor comprising adaptor protein apoptosis-associated speck-like protein containing a CARD (ASC) upstream sensor protein NLRP3, and the effector protein Casp-1 (caspase-1). NLRP3 inflammasome activation results in active Casp-1 cleaving and subsequent release of interleukin-1β (IL-1β) (Zhang et al., 2016, Liu et al., 2019). Furthermore, activate Casp-1 induces the formation of GSDMD-N (N-terminal domain of gasdermin D) which induces pyroptosis and provokes the inflammatory cytokines release that activate the recruitment of polymorphonuclear neutrophils (PMNs) to the inflammation site causing “respiratory burst”, overproduction of ROS, and amplification of the cytokine storms. Many studies demonstrated that NLRP3 inflammasome inactivation exerted a protective influence against septic ALI (Zhang et al., 2021, Kang et al., 2022, Zhang et al., 2022).

Oxidative stress is fundamental in the pathogenesis of ALI/ARDS. Excessive generation of oxidants as ROS has direct damaging effects on the cellular components. Furthermore, some oxidants can act as inflammatory signal molecules to activate major inflammatory pathways as NF-κB and NLRP3 inflammasome leading to exacerbation of the inflammatory response (Li et al., 2020). Endogenous antioxidants such as superoxide dismutase (SOD), catalase (CAT), and reduced glutathione (GSH) target to eliminate the harmful oxidants. Nrf-2 (nuclear-factor erythroid-2-related factor) is the main signal transcription factor that regulates antioxidant substances in the body. Normally, Keap-1 (Kelch-like-ECH-associated protein-1) captures Nrf2 in the cytoplasm. Once activated by stressful initiators, Nrf2 disconnects from Keap-1 and moves to the nucleus where it actuates the antioxidant response elements (AREs) as antioxidant enzymes (GSH, SOD, CAT) as well as antioxidant genes (HO-1/GCL/NQO-1). Activation of Nrf-2 attenuates oxidative stress and inflammatory response and so confer protection against experimental ALI (Liu et al., 2018; 2017; Wu et al., 2021).

Various natural metabolites exhibit multiple anti-inflammation and lung protective effects, such as alkaloids, flavonoids, and terpenoids (El-Agamy et al., 2020, He et al., 2021; Mohamed et al., 2021). Flavonoids are widely found in fruits, vegetables, and various plants. Many of these flavonoids are reported to weaken inflammatory responses through diverse mechanisms (Tauchen et al., 2020, He et al., 2021). Among these flavonoids, kaempferol and its glycosides gained a growing interest because of potential diverse uses as functional foods and food supplements, as well as in cosmetic and pharmaceutical preparations. Numerous pharmacological effects of kaempferol and its glycosylated derivatives have been documented, including neuroprotective, cardioprotective, antidiabetic, anti-inflammatory, antioxidant, and anticancer activities (Devi et al., 2015, Alkandahri et al., 2023). Notably, previous studies have demonstrated the safety of kaempferol and its glycosylated derivatives (Yangzom et al., 2022, Alkandahri et al., 2023, Lee et al., 2023). Interestingly, a recent study by Akiyama et al. (2023) has shown the safe use of kaempferol aglycone to healthy adults (Akiyama et al., 2023). Kaempferol 3-sophoroside-7-glucoside (KSG) is one of the major flavonol reported from saffron (Moratalla-López et al., 2019). It was also separated from *Brassica* spp., *Lycium chinense*, *Hosta ventricose,* and *Equisetum debile* (Calderón-Montaño et al., 2011, Choi et al., 2019)*.* Little data regarding the pharmacological activities of KSG is available. However, a recent study reported the potential *in-vitro* anti-inflammatory and antioxidant activities of KSG (He et al. 2023). An additional study by Lee et al. revealed that the KSG-rich roasted *Lycium chinense* (goji berry) leaves extract did not show any signs of toxicity, pathological abnormalities, or death to rats, and the ALD (approximate lethal dose) was found to be greater than 2000 mg/kg (Lee et al., 2023).

Molecular docking is a computer-based technique crucial in the drug discovery process. It anticipates how ligands will position themselves within the active sites of receptors, relying on their three-dimensional structures (Omar et al., 2022, Omar et al., 2023). The primary objectives of molecular docking are to uncover the binding mode of a ligand to a receptor and to assess the strength of their interaction. Molecular dynamics (MD) serves as a computational simulation method aimed at comprehending the structure and function of molecules. By predicting the movements of each atom in a molecular system over time, MD simulations rely on a general model of physics governing atom interactions (Alzain et al., 2023, Shoaib et al., 2023). These simulations provide insights into various biomolecular processes, including conformational changes, ligand binding, and protein folding. Importantly, they offer a femtosecond temporal resolution, revealing the precise positions of all atoms during these dynamic processes.

The present study explored the effects of KSG against ALI and its implicit molecular pathways that are related to repression of the inflammatory response, oxidative stress, and apoptotic changes using various experimental and computational methods.

## 2 Materials and methods

### 2.1 Materials

Kaempferol 3-sophoroside-7-glucoside (KSG) was purchased from Chengdu Biopurify Phytochemicals Ltd, China and dissolved in saline*.* Lipopolysaccharide (LPS, Escherichia coli serotype O111:B4) was obtained from Sigma Aldrich, USA.

### 2.2 Animal experiments

Male Balb/c albino mice (25 to 30 g) were obtained from medical research center (MERC) (Faculty of Medicine, Mansoura University) and acquainted under standard 12 h dark/light cycles, temperature, and humidity conditions for one week before the conduction of the experiment. The experimental protocol has been approved from Research Ethics Committee, King Abdulaziz University, KSA (approval number: PH-116–40).

### 2.3 ALI model

Establishment of ALI model was based on the previous study (Mohamed et al., 2021; Abdallah et al., 2020). Mice were injected once with LPS (10 mg/kg/I.P.). Six groups of six mice each were randomly assigned to receive the following treatments: (1) Mice in the control group received vehicle for five days.; (2) KSG 100, mice received KSG (100 mg/kg, oral once daily/5 days); (3) LPS, mice were administered LPS I.P.; (4) KSG 50 + LPS and (5) KSG 100 + LPS, mice received KSG (50 and 100 mg/kg, respectively, oral/once daily/ 5 days before LPS injection and (6) DEXA + LPS, mice received dexamethasone (5 mg/kg/once daily/5 days) before LPS injection and this group served as positive control group. Doses of KSG were selected based on previous data of relevant kaempferol derivatives that showed potent anti-inflammatory activities (Rabha et al., 2018, Chen et al., 2020). Mice were anesthetized using xylazine/ketamine (10/75 mg/kg, respectively) and executed humanely by cervical dislocation 24 h following LPS injection. Lungs were excised for the following estimations.

### 2.4 Histopathology

The left lung was fixed in formalin for 24 h and inserted in paraffin before sectioning (4–5 µm). Lung specimens were stained with hematoxylin/eosin (H/E). Pulmonary injury was measured blindly by two pathologists as formerly stated (El-Agamy et al., 2020, Li et al., 2020). and recorded based on the existence of alveolar congestion; hemorrhage; inflammatory cell infiltration; and thickness of the alveolar wall. Score 0 = normal, 1 = mild injury (<25 %), 2 = moderate (25–50 %), 3 = severe (50–75 %) and 4 = very sever (>75 %).

### 2.5 Immunohistochemistry (IHC)

IHC staining for TNF-α, NF-κB, Casp-1, and Nrf2 was implemented automatically utilizing a BenchMark Ventana-XT system (Ventana- Medical Systems/USA) as described previously (Mohammed et al., 2021). Software for digital image analysis was used to conduct semi-quantitative estimation (ImageJ, 1.46a; NIH).

#### 2.5.1 W/D (Lung wet/dry weight) ratio

W/D ratio was employed to identify pulmonary edema. For estimating dry weight (D), a small piece of the left lung was weighed (W) and heated for 24 h in an oven (80 °C) (El-Agamy et al., 2020; Mohammed et al., 2021). The W/D ratio was estimated.

#### 2.5.2 BALF (Bronchoalveolar lavage fluid)

BALF was obtained as stated earlier (Rizq et al., 2023). The lungs were exposed after opening the chest. The left lung was clamped before perfusing the right lung with sterile saline three times. BALF was collected and centrifuged to get the supernatants that were stored at −80℃ for further analysis while cell pellets were used for the determination of the cell counts and myeloperoxidase (MPO) activity.

#### 2.5.3 Protein content and LDH activity

In BALF, LDH activity and protein content were estimated according to the kits’ protocol (Human/Germany and Thermo Fisher Scientific/USA, respectively) using a spectrophotometer (UNICO Instruments C., Model 1200 USA).

#### 2.5.4 Counts of inflammatory cells

A hemocytometer was utilized to estimate the total and differential cell counts in the BALF. After suspending the cell pellets in saline 0.9 %, they were centrifuged onto slides and stained for 8 min with Wright/Giemsa. Quantification of differential cell counts were done at 40 × magnification through counting a total of 200 cells/slide by light microscope. Each cell type number was calculated as % of cell type × total number of cells in the BALF (Rizq et al., 2023).

#### 2.5.5 MPO activity

It was estimated as early reported (Shaaban et al., 2018). Briefly, phosphate buffer was added to cell pellets and centrifuged. Pellets were resuspended in sodium phosphate buffer containing hexadecyltrimethylammonium bromide and the suspension was frozen and thawed for 3 cycles. The suspension was kept at 60 °C for 2 h and then centrifuged to get the supernatant. Finally, sodium-phosphate buffer containing H_2_O_2_ and tetramethylbenzidine was added to the supernatant. Absorbance change at 650 nm was registered for 5 min.

#### 2.5.6 ELISA (Enzyme-linked immunosorbent assay)

The levels of NF-κBp65, TNF-α, IL-1β, NLRP3, GSDMD-N, and HO-1 were estimated in the lung supernatants utilizing ELISA kits following the manufacturer's specifications (MyBioSource/USA, R&D Systems/USA). The absorbance was measured using ELISA plate reader (Tecan, Sunrise Absorbance Reader/Austria). Casp-1 was estimated colorimetrically using a commercial kit (BioVision/USA). In the nuclear extracts, Nrf2-binding capacity was estimated based on the kit's protocol (Active Motif/USA).

#### 2.5.7 Lipid peroxidative and antioxidant markers

Lipid peroxidative parameters [MDA (malondialdehyde), PC (protein carbonyl), and 4-HNE (4-hydroxynonenal)], as well as antioxidants [GSH, SOD, and CAT] were determined in the supernatants of the lung homogenates using commercial kits (MyBioSource/USA; Bio-diagnostic Co./Egypt).

#### 2.5.8 RT-PCR

The gene expression analysis was performed according to the kits’ instruction. Briefly, extraction and quality testing of total RNA were done utilizing RNeasy Mini kit and non-pure samples were discarded. Reverse transcription of RNA into cDNA was carried out and followed by quantitative RT/PCR using SYBR Green and the primers sequences for the target gene listed in Table S1. The ^ΔΔ^Ct technique was used to calculate the relative amounts of target mRNAs after normalizing them to β-actin.

#### 2.5.9 Survival rate

Another 4 groups (n = 10) of mice were assigned and treated as follows: control, KSG 100, LPS, and KSG 100 + LPS. KSG (100 mg/kg) was given for 5 days once daily before LPS challenge (10 mg/kg I.P.). The animals were maintained for 3 days under monitoring lethality every 6 h. According to the death number, the survivor percentage was computed.

#### 2.5.10 Statistical analysis

One-way ANOVA, then Tukey's Kramer multiple comparisons Tests were used to compare the various groups. Data are the means ± SE (n = 6). Significant difference was adopted when *P* value < 0.05.

### 2.6 Computational studies

The *in-silico* investigations in this study utilized Maestro software package version 12.8 developed by Schrödinger Inc. The Molecular Dynamics (MD) simulations were performed using the Academic version of Desmond, software developed by D.E. Shaw Research.

#### 2.6.1 Protein preparation

The KEAP1 kelch domain crystal structure (PDB ID: 7OFE) in complex with inhibitor VBQ was obtained from the RCSB Protein Data Bank with a resolution of 1.19 Å. The Maestro software, utilizing the OPLS4 forcefield, was employed to optimize and minimize the protein structure. Standard procedures, such as removing water molecules and heteroatoms, adding missing hydrogen atoms, and adjusting bond orders, were carried out (Khedr et al., 2024). A restrained minimization with a default RMSD value of 0.30 Å was applied to refine the protein structure.

#### 2.6.2 Ligand preparation

The structure of KSG (CID: 12960459) was retrieved from the PubChem database. Ligprep module of the Maestro was utilized to prepare the ligand, generating energetically minimized 3D conformers with different stereoisomers, conformations, tautomers, and ionization states, while retaining lead-like compounds (Ibrahim et al., 2022).

#### 2.6.3 Receptor grid generation

A 3D grid representing the binding region of KEAP1 was generated using the bound ligand in the Glide module of the Maestro. This grid was employed in subsequent molecular docking studies.

#### 2.6.4 Molecular docking and MM-GBSA calculations

Glide module of the Maestro was used for docking the prepared ligand into the active site of KEAP1. Glide evaluates binding affinities and generates scoring functions. The Extra Precision (XP) docking mode was employed for the docking process, requiring prepared protein and ligands, along with 3D grid files representing the active site properties of the receptor. Relative MM-GBSA binding affinity between KEAP1 and the ligand was assessed by calculating the binding free energies of the complex using the Prime module of the Maestro. These binding free energies served as an indicator of the thermodynamic stability between the ligand and the target.

#### 2.6.5 Molecular dynamics (MD) simulations

MD simulations were conducted using Academic Desmond v6.5 to predict the stability of the docked complex over 100 ns (Ibrahim et al., 2022, Alzain et al., 2024). The solvated system, built with the System Builder panel and utilizing the OPLS4 forcefield, included water molecules with a TIP3P model in a buffered orthorhombic box. The system was neutralized with Na^+^ and Cl^-^ ions (0.15 M concentration) to maintain physiological conditions. The NPT ensemble method was employed at a constant temperature of 300 K and pressure of 1 bar. The resulting trajectory from MD simulations was analyzed for RMSD, RMSF, and other molecular interactions to evaluate the stability of the docked complex.

## 3 Results

Regarding all estimated parameters, there was no apparent difference between the KSG control group and the control group.

### 3.1 KSG improved survival time and repressed LPS- induced ALI

**Fig. 1:**
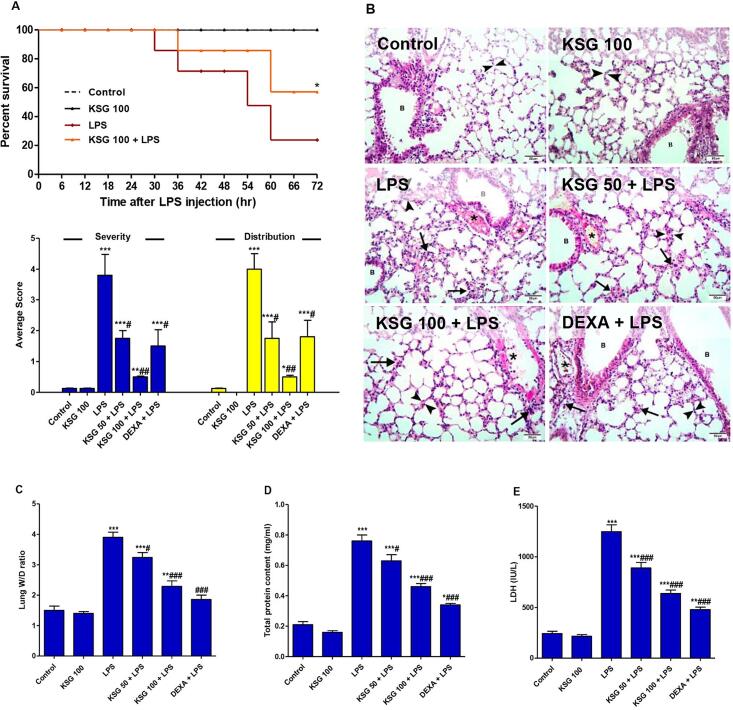
**KSG improved survival time and repressed LPS- induced ALI.** A. Survival rate of LPS-intoxicated mice. B. Histopathological analysis of lung specimen stained with H&E stain × 40, scale bar 50 µm. The control and KSG sections showed normal structure of respiratory bronchioles (B) with this interalveolar septae (between arrow heads). LPS group: showed vascular congestion (asterix), interalveolar hemorrhage, inflammatory cell infiltration (arrows) with oedema and thickening of the interalveolar septae (between arrow heads). The respiratory bronchioles (B) are lined with disturbed thickened epithelial lining. In the KSG + LPS groups, the LPS-produced lesions remarkably improved that was more prominent in the group receiving higher dose of KSG compared to DEXA pretreated group which exhibited moderate lesions, Pathological alterations in lung tissue were evaluated semi-quantitatively across all groups; C. Lung W/D ratio; D. Total protein content; E. LDH (Lactate dehydrogenase) activity in BALF. Values are the mean ± SEM (n = 6). * P < 0.05, ^**^ P < 0.01, ^***^ P < 0.001 vs control group; ^#^ P < 0.05, ^##^ P < 0.01, ^###^ P < 0.001 vs LPS group (one-way ANOVA).

LPS injection notably lowered the survival rate compared to the normal animals. Compared to the LPS group, the pre-treatment with KSG considerably raised the survival rate (Fig. 1A). LPS injection resulted in ALI that was evident through the significant lung lesions and elevated parameters of injury. Histopathological analysis of the lung tissue revealed the normal lung architecture of the control mice with no sign of any pathological lesion (Fig. 1B). LPS challenge instigated widespread injury in the form of inflammatory cell infiltration, interalveolar hemorrhage, vascular congestion, oedema and thickening of the interalveolar septae. Besides histopathology, the noticeable pulmonary edema as the protein content of BALF and lung W/D ratio were elevated in LPS group, compared to control (Fig. 1C, D). Also, LPS induced high LDH activity in BALF (Fig. 1E). Interestingly, KSG pretreatment reversed all the observed LPS-induced injurious effects. The lung lesions were greatly alleviated especially in the group receiving the higher dose of KSG (100 mg/kg) and the lesions’ scores were notably attenuated. The effect of KSG was nearly comparable to DEXA. Also, protein content, lung W/D ratio, and LDH activity were all depressed in the KSG pretreated groups.

### 3.2 KSG attenuated inflammatory cell infiltration in LPS- induced ALI

**Fig. 2:**
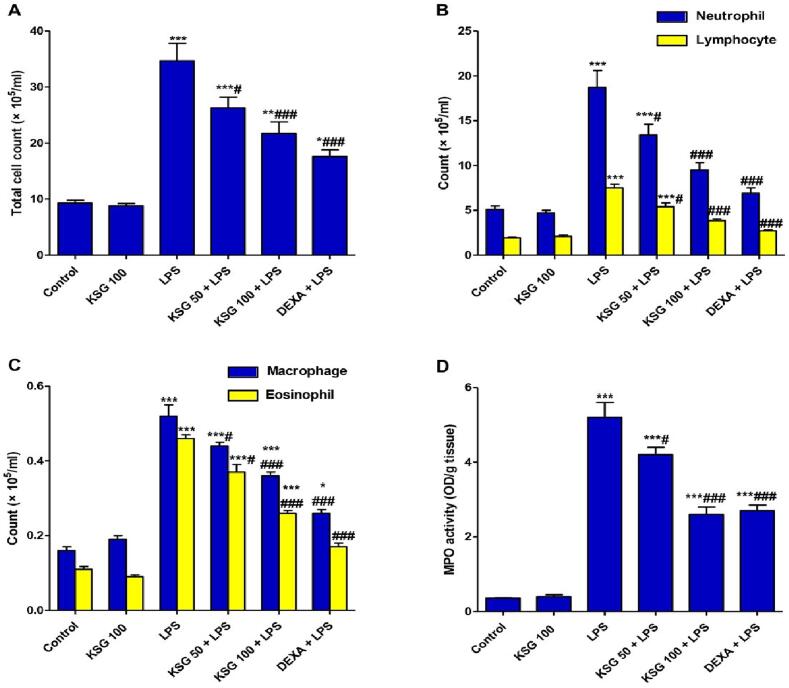
**KSG reduced inflammatory cell infiltration caused by LPS.** A-C Total and differential cell count; D. MPO activity in lung tissue. Values are the mean ± SEM (n = 6). ^**^ P < 0.01, ^***^ P < 0.001 vs control group; ^#^P < 0.05, ^##^P < 0.01, ^###^P < 0.001 vs LPS-group (one-way ANOVA).

Another important parameter of lung lesion was the intensified inflammatory cell infiltration into the pulmonary tissue in LPS group. When compared to control mice, the total and differential cell counts in BALF were considerably higher (Fig. 2). Additionally, MPO activity was boosted in LPS group which is a known index for neutrophil infiltration. On the contrary, LPS-boosted inflammatory cell infiltration was ameliorated and MPO level was decreased in groups that had received KSG pretreatment.

### 3.3 KSG reduced NF-κB/TNF-α signaling activation induced by LPS

**Fig. 3:**
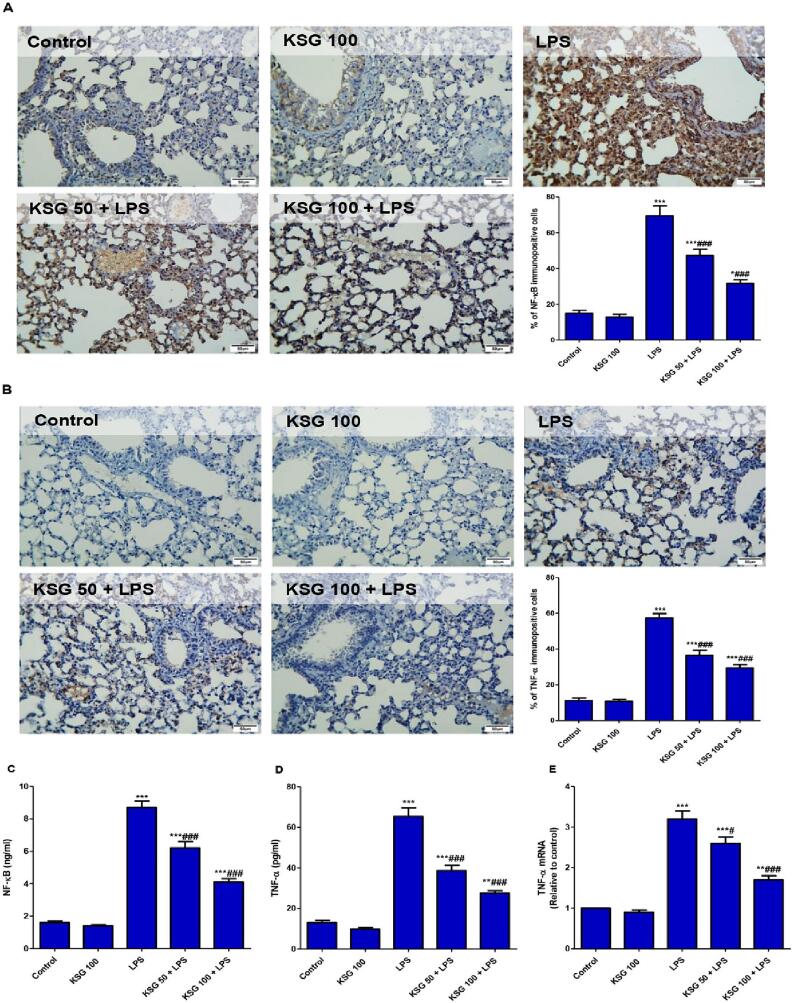
**KSG repressed LPS-associated activation of NF‑κB/TNF-α signalling/cytokine release in lung.** A&B. IHC analysis of NF‑ĸB and TNF-α in lung tissue, respectively. Control and KSG groups, the positive NF‑ĸB/TNF-α staining was minimal; LPS group, there was increased expression of NF‑ĸB/TNF-α‑positive cells; KSG 50 + LPS group, there was lower degree of NF‑ĸB/TNF-α positive staining; KSG 100 + LPS group, NF‑ĸB/TNF-α positive staining was minimal; Semiquantitative analysis expressed as % of immunopositive cells. C&D. Level of NF‑κB and TNF‑α respectively. E. mRNA expression of TNF‑α. Values are the mean ± SEM (n = 6). ^**^ P < 0.01, ^***^ P < 0.001 vs control group; ^#^ P < 0.05, ^###^ P < 0.001 vs LPS group (one-way ANOVA).

As presented in Fig. 3, LPS injection significantly elevated the immunoexpression of NF-κB and TNF-α as well as level of NF-κB compared to control mice. The level as well as the gene expression of TNF-α were also enhanced in LPS group compared to control one. Contrarily, KSG pretreated groups showed notable reduction of immunoexpression and level of NF-κB and TNF-α as well as the gene expression of TNF-α compared to LPS group.

### 3.4 KSG abrogated LPS-induced activation of NLRP3/Casp-1/IL-1β/GSDMD signaling

**Fig. 4:**
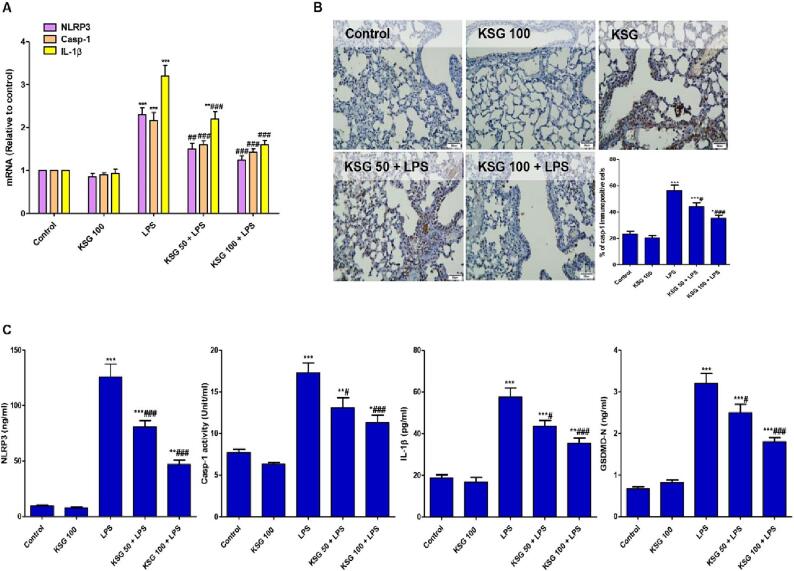
**KSG suppressed LPS-induced activation of NLRP3/Casp-1/ IL-1β signaling axis.** A. mRNA expression of NLRP3/Casp-1/IL-1β. IHC analysis of lung specimen stained for Casp-1: Control and KSG groups exhibited minimal stain for Casp-1 where LPS group showed increased expression of Casp-1 positive cells; KSG 50 + LPS and KSG 100 + LPS groups showed decreased immunostain of Casp-1; Semiquantitative analysis expressed as % of immunopositive cells. C. Levels of NLRP3/Casp-1/IL-1β/GSDMD-N. Values are the mean ± SEM (n = 6). * P < 0.05, ^**^ P < 0.01, ^***^ P < 0.001 vs control group; ^#^ P < 0.05, ^##^ P < 0.01, ^###^ P < 0.001 vs LPS group (one-way ANOVA).

The inflammatory pathway of NLRP3 is enhanced following LPS injection. The mRNA expression and the levels of NLRP3/Casp-1/IL-1β were enhanced in LPS group compared to control one. Additionally, the immunoexpression of Casp-1 was heightened as well as GSDMD-N was significantly elevated after LPS injection. Contrarily, KSG pretreated groups exhibited significant amelioration of the above-mentioned parameters (Fig. 4).

### 3.5 KSG enhanced Nrf2 cascade signaling

**Fig. 5:**
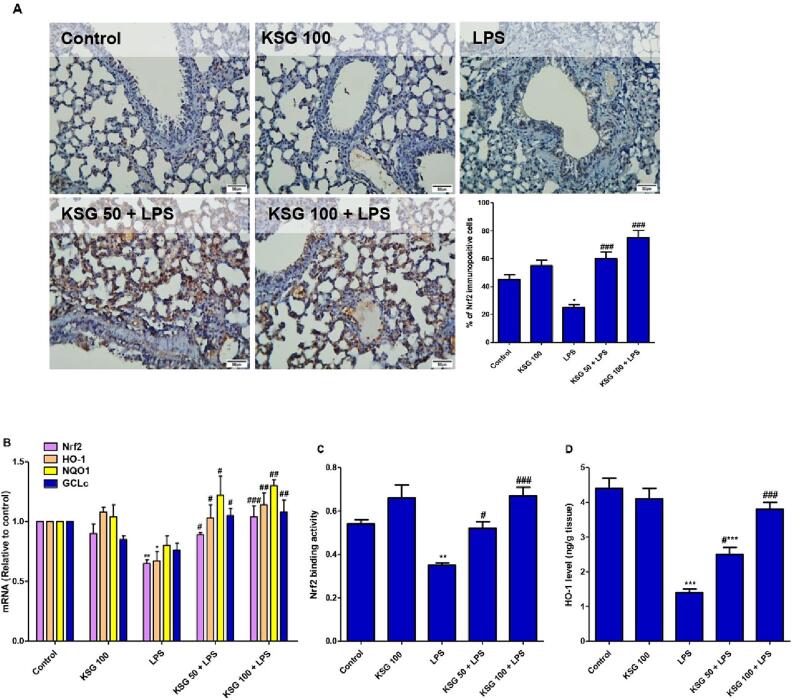
**KSG enhanced Nrf2 signaling in lung of LPS-intoxicated mice.** A. IHC analysis of lung specimen immunostained for Nrf2; B. mRNA expression of Nrf2, HO-1, NQO1, and GCLc; C. Nrf2 binding activity; D. HO-1 level. Values are the mean ± SEM (n = 6). * P < 0.05, ^**^ P < 0.01, ^***^ P < 0.001 vs control group; ^#^ P < 0.05, ^##^ P < 0.01, ^###^ P < 0.001 vs LPS group (one-way ANOVA).

LPS injection resulted impairment of Nrf2 signaling as it reduced the immunoexpression of Nrf2 as well as mRNA expression of Nrf2/HO-1/NQO1/GCLc compared to control animals. In addition, LPS lowered the binding activity of Nrf2 and decreased HO-1 level in lung tissue. On the other hand, KSG pretreatment opposed LPS-induced changes in Nrf2/HO-1 signaling as it enhanced its genetic and protein expression concurrent with elevation in its binding activity (Fig. 5).

### 3.6 KSG attenuated LPS-caused lipid peroxidation and enhanced antioxidants

As demonstrated in Table 1, there was a significant rise in the lipid peroxidative markers, MDA, PC and 4-HNE, in the lung tissue of LPS group compared to control one. Simultaneously, there was a significant decrease in the antioxidants (GSH, SOD, CAT) compared to control group. Pretreatment with KSG notably lessened the lipid peroxidative parameters and increased the antioxidants compared to LPS group.

**Table 1:** KSG attenuated LPS-induced lipid peroxidation and enhanced antioxidants.

**Parameters**	**Groups**					
	**Control**	**KSG 100**	**LPS**	**KSG (50 mg/kg) + LPS**	**KSG (100 mg/kg) + LPS**	**DEXA + LPS**
**MDA (nmol/g tissue)**	23.1 ± 2.1	20.9 ± 1.7	65.9 ± 5.8 ^***^	49.9 ± 3.7 ^***#^	39.8 ± 2.4 ^*###^	33.4 ± 1.7 ^*###^
**PC (nmol/mg tissue)**	0.51 ± 0.03	0.46 ± 0.02	1.62 ± 0.1 ^***^	1.37 ± 0.06 ^***#^	1.02 ± 0.14 ^**##^	0.82 ± 0.02 ^*###^
**4-HNE (µmol/ml)**	0.66 ± 0.02	0.54 ± 0.04	1.87 ± 0.09 ^***^	1.43 ± 0.06 ^***#^	1.19 ± 0.12 ^***##^	1.12 ± 0.09 ^**##^
**GSH (µmol/g tissue)**	11.5 ± 0.6	10.21 ± 1.2	4.6 ± 0.33 ^***^	7.8 ± 0.6 ^*#^	9.11 ± 0.8 ^###^	8.1 ± 0.64 ^###^
**SOD (U/g tissue)**	37.1 ± 1.2	38.3 ± 2.8	9.3 ± 0.2 ^***^	19.1 ± 1.7 ^**##^	29.9 ± 2.1 ^###^	26.5 ± 1.6 ^###^
**CAT (ng/g tissue)**	44.3 ± 4.3	47.5 ± 1.6	18.6 ± 1.5 ^***^	29.4 ± 2.8 *	36.2 ± 3.5 ^##^	30.2 ± 2.3 ^*#^

Malondialdehyde (MDA); protein carbonyl (PC); 4-hydroxynonenal (4-HNE); reduced glutathione (GSH); superoxide dismutase (SOD); catalase (CAT). Values are the mean ± SEM (n = 6). *P < 0.05, ^**^P < 0.01, ^***^P < 0.001 vs control group; ^#^ P < 0.05, ^##^ P < 0.01, ^###^P < 0.001 vs LPS-group (one-way ANOVA).

### 3.7 Computational studies

#### 3.7.1 Molecular docking and MM-GBSA calculations

**Fig. 6:**
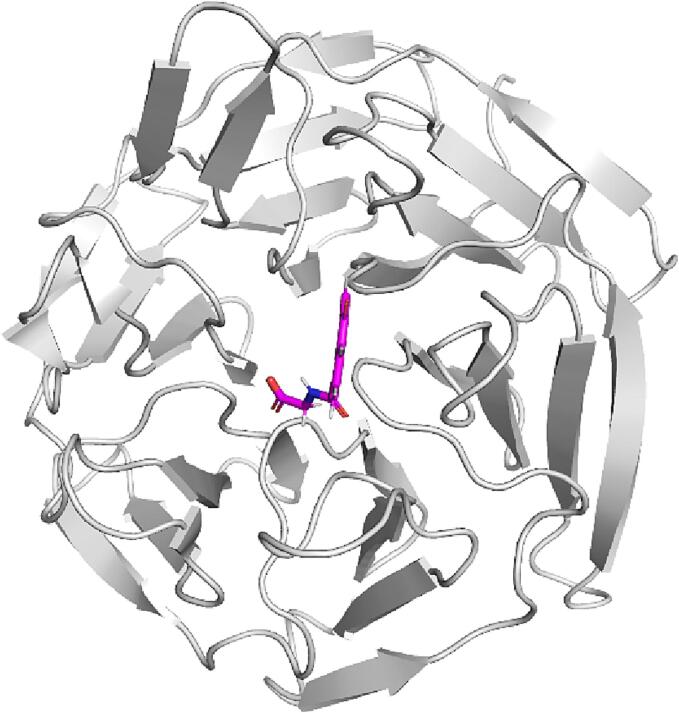
Crystal structure of KEAP1 bound with the reference (PDB ID: 7OFE).

To determine and evaluate the interactions between KSG and KEAP1 protein and determine plausible inhibition mechanism of Keap1-Nrf2 protein–protein interactions, molecular docking study of the ligand and the reference were conducted against KEAP1 (PDB ID: 7OFE) (Fig. 6). The RMSD value of the co-crystallized ligand and the re-docked ligand was 0.7199 Å indicates the accuracy of the docking method utilized in this research.

Table 2 summarizes the docking scores and MM-GBSA binding free energies of Kaempferol 3-sophoroside-7-glucoside (KSG) and the reference inhibitor with KEAP1. KSG exhibited a superior docking score of −9.576 Kcal/mol compared to the 7OFE reference inhibitor, which had a score of −6.633 Kcal/mol. Additionally, the MM-GBSA binding free energy calculations further highlighted the favorable binding affinity of KSG, with a value of −67.25 Kcal/mol, surpassing the reference inhibitor at −56.36 Kcal/mol.

**Table 2:** Docking scores, MM-GBSA binding free energies of KSG and the reference with KEAP1 (PDB ID: 7OFE).

**Compound**	**Docking scores Kcal/mol**	**MM-GBSA dG bind Kcal/mol**
KSG	−9.576	−67.25
7OFE reference	−6.633	−56.36

**Fig. 7:**
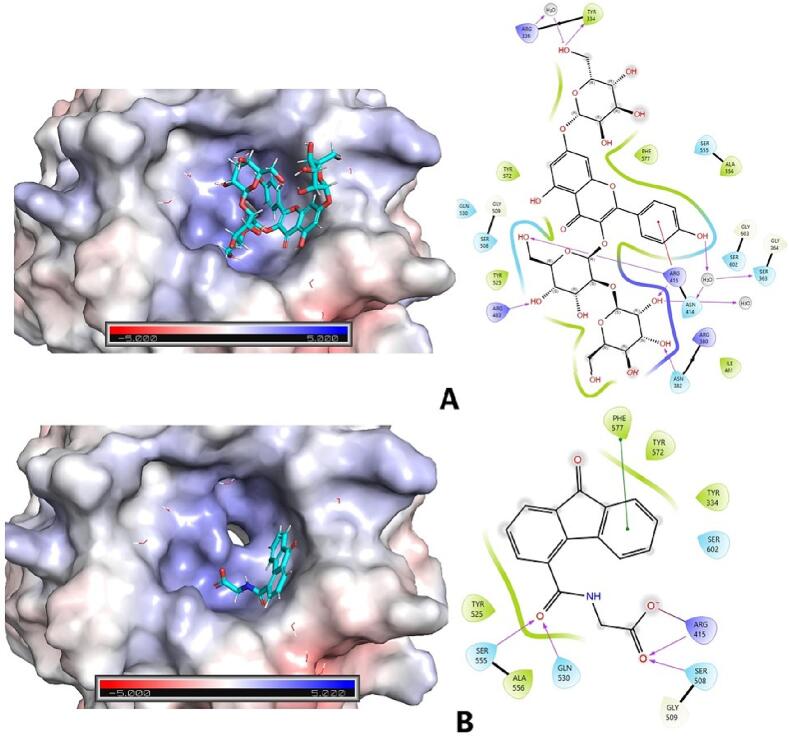
3D and 2D interactions of KSG and the reference with KEAP1 (PDB ID: 7OFE). KSG, (B) Reference.

In examining the molecular interactions of KSG within the KEAP1 binding site, we observed several noteworthy patterns (Fig. 7).

Hydrogen bonds were identified with TYR334, ASN382, ARG415, and ARG483, indicating robust interactions at these specific locations. Additionally, KSG engaged in water bridge interactions with ARG336, SER363, and ASN414, contributing to further stabilization within the binding pocket. A distinctive Pi-pi interaction between KSG and PHE577 underscored an aromatic interaction within the binding site. Furthermore, hydrophobic interactions with TYR334, TYR525, ALA556, TYR572, and PHE577 highlighted the involvement of hydrophobic residues in facilitating ligand-receptor interactions. The molecular interactions of the reference inhibitor within the KEAP1 binding site exhibited hydrogen bonds with ARG415, SER508, GLN530, and SER555. Notably, a Pi-cation interaction was identified between the reference inhibitor and ARG415. Similar to KSG, the reference inhibitor engaged in hydrophobic interactions with TYR334, TYR525, ALA556, TYR572, and PHE577, underscoring the significance of hydrophobic contacts in ligand binding.

#### 3.7.2 Molecular dynamics studies

In this study, we employed the Academic Desmond to explore the dynamic interactions within docked complexes. Molecular Dynamics (MD) simulations were conducted on KSG and a co-crystal ligand with the KEAP1 protein (PDB ID: 7OFE). The primary objectives were to investigate the binding mode, assess the stability of the protein–ligand complex, and analyze the interaction patterns. The MD simulations involved a thorough examination of ligand behavior over a period of 100 ns, generating a total of approximately 1000 frames at intervals of 100 picoseconds along the trajectory.

**Fig. 8:**
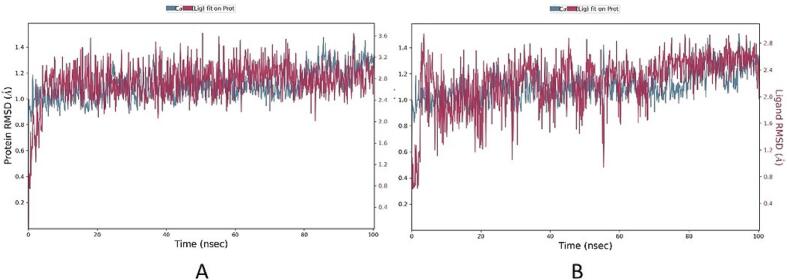
RMSD plot of KSG and the reference with KEAP1 (PDB ID: 7OFE) during 100 ns MD simulations. (A) KSG, (B) Reference.

To explore the stability of the docked complexes, we utilized Root Mean Square Deviation (RMSD), a metric illustrating structural variability and protein stability. The RMSD outcomes below 1.4 Å for KSG and the co-crystal ligand with KEAP1 indicate the stability of the complexes during MD simulations (Fig. 8).

**Fig. 9:**
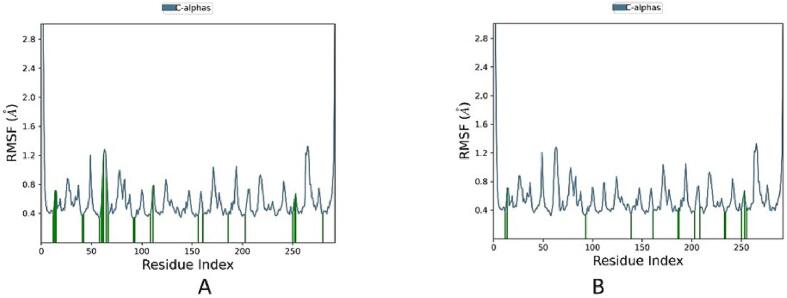
Protein RMSF plot of KEAP1 bound to KSG and the reference (PDB ID: 7OFE) during 100 ns MD simulations. (A) KSG, (B) Reference.

Throughout the 100 ns of simulation, there was no observable change in the Root Mean Square Fluctuation (RMSF) of the protein–ligand complex, as depicted in Fig. 9. Additionally, both KSG and the co-crystal ligand exhibited comparable stability patterns, maintaining RMSF values consistently below 1.3 Å throughout the entire MD simulation study.

**Fig. 10:**
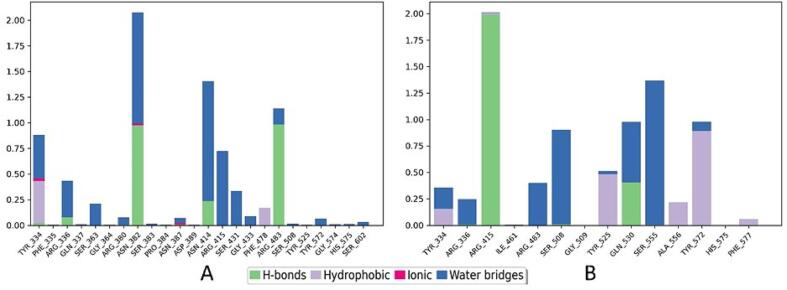
Histogram interaction of KSG and the reference with KEAP1 (PDB ID: 7OFE) during 100 ns MD simulations. (A) KSG, (B) Reference.

In the MD studies, the interactions of KSG within the binding site revealed noteworthy patterns (Fig. 10). TYR334 played a dual role with a predominant 41 % hydrophobic and 42 % water bridge interaction with KSG. Additionally, ASN382 exhibited a substantial 98 % hydrogen bond interaction and 101 % water bridge interaction with KSG. ASN414 demonstrated a remarkable 117 % water bridge interaction Moreover, ARG415 exhibited a notable 72 % water bridge interaction. Finally, ARG483 displayed a significant 98 % hydrogen bond interaction with KSG.

In comparison, the reference inhibitor showcased distinct molecular interaction patterns. ARG415 demonstrated 200 % hydrogen bond interaction. ARG483 exhibited a moderate 40 % water bridge interaction. SER508 displayed an 89 % water bridge interaction. TYR525 contributed significantly with a 48 % hydrophobic interaction. GLN530 displayed versatility with a 48 % hydrogen bond interaction and a 57 % water bridge interaction. SER555 exhibited a notable 136 % water bridge interaction. Lastly, TYR572 demonstrated a significant 89 % hydrophobic interaction, underscoring its significant contribution to hydrophobic contacts.

## 4 Discussion

**Fig. 11:**
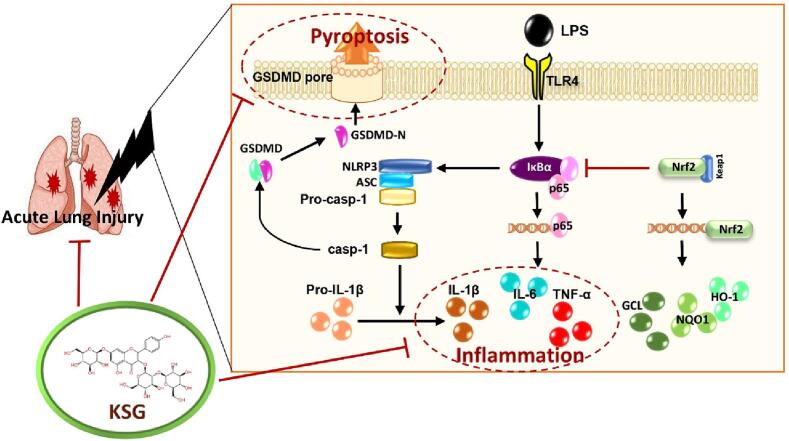
The possible molecular mechanisms that mediate the protective activity of KSG against LPS-induced ALI.

Despite current efforts, the pathogenesis of ARDS/ALI is obscure and still lacks innovative therapies. Nevertheless, the dysregulated inflammation response with resultant impairment of gas exchange plays a critical role in the pathophysiology of septic ALI. Exploring new potent anti-inflammatory agents represents a promising research orientation. Results of the present study, summarized in the graphical (Fig. 11), proved that KSG pre-treatment could improve the survival of LPS-intoxicated mice and attenuates LPS-induced ALI. The protective activity of KSG could be attributed to suppression of the inflammatory response and pyroptosis via modulation of the inflammatory axis NF-κB/NLRP3/GSDMD. Furthermore, KSG potentiated the antioxidant capacity and enhanced the Nrf2 signaling in the lung tissue of septic mice. In summary, KSG is a promising therapeutic agent for protection against ALI in septic mice through modulation of pro-inflammatory and anti-oxidative machinery (Fig. 11).

LPS, a bacterial cell wall component, has been widely employed for establishing an ALI mouse model. Administration of LPS results in sever inflammatory response and ALI within hours (Shaaban et al., 2018). Our results have shown that LPS induced ALI in mice that was evident through notable pathological lesions in HE-stained lung specimen. Furthermore, increase in lung W/D ratio and the total protein content of BALF indicated the existence of pulmonary edema that happens due to excessive infiltration of activated neutrophils and macrophages. Activated infiltered inflammatory cells increase the capillary and alveolar permeability leading to the exudation of proteins from plasma (Zhang et al., 2018, El-Agamy et al., 2020). The damage of lung cellular integrity was confirmed through the LDH high level in BALF. Interestingly, the LPS-induced pathological and biochemical changes were lightened in the KSG pretreated groups.

Early inflammatory response during LPS-induced ALI include the activation and inflammatory cells accumulation, particularly, neutrophils in lung. Increased neutrophil not only participates in pulmonary edema development but also in the amplification of the inflammatory response by recruiting more leukocytes (Abdallah et al., 2020; Mohamed et al., 2021). Furthermore, neutrophil enhances immunological response and chronic inflammation through the release of IL-17 which in turn effectuates pro-inflammatory mediators release such as metalloproteinases, cytokines, and chemokines. MPO is a major constituent of neutrophil cytoplasmic granules, and its high level is a direct measure of the accumulation of activated neutrophils and reflects lung injury (El-Agamy, 2011; Ibrahim et al., 2019). Our results have shown a remarkable increase in the differential and total cell counts in BALF in the LPS group as well as a remarkable increase in the MPO level confirming the accumulation of neutrophil in lung tissue. On the contrary, KSG pretreatment attenuated the inflammatory cells infiltration into lung tissue along decreased MPO level. Collectively, these results confirm that KSG exhibited remarkable protective effect against LPS-induced ALI. It is worth mentioning that previous reports have shown that other kaempferol aglycone derivatives and apigenin-7-glucoside have protective efficacy against LPS-induced ALI (Chen et al., 2012, Li et al., 2015, Qian et al., 2019). Furthermore, diosmin downregulated NF-κB activation and the expression of pro-inflammatory cytokines in septic mice (Imam et al., 2015).

In a next step, the possible molecular mechanisms that mediate the protective activity of KSG against LPS were investigated. To be precise, oxidative stress and inflammation were the primary considerations to explore.

LPS-mediated excessive inflammation has many pathogenic factors. LPS binds to TLR4 (toll-like receptor-4), a pattern recognition receptor, in the lung tissue transducing signals to activate several signaling pathways including NF-κB. Activation of NF-κB transcription factor mediates the induction and expression of inflammatory cytokines like ILs and TNF-α (Wu et al., 2021, Cai et al., 2022, Liu et al., 2022). In addition, stimulation of monocytes and macrophages by LPS induces the production of TNF-α which is multifunctional cytokine. It generates an inflammatory cascade to release other cytokines and chemokines like IL-6 that recruit more polymorphonuclear cells which cause further damage to the tissue (El-Agamy et al., 2020). Our results confirmed LPS-induced activation of NF-κB as LPS injection significantly enhanced NF-κB signaling and increased the levels of downstream cytokine, TNF-α in the lung tissue. Notably, KSG was found to effectively inhibit the progression of inflammation. KSG pretreatment inhibited NF-κB activation and so attenuated the increase in the expression and level of TNF-α.

In addition to NF-κB, NLRP3 inflammasome is another key inflammatory mediator that has been implicated in LPS-induced ALI. Activation of NLRP3, under oxidative and inflammatory conditions, leads to the maturation and overproduction of inflammatory cytokine, IL-1β which participates in the development of ALI. NLRP3-related pyroptosis is implicated in the LPS-stimulated ALI pathogenesis (He et al., 2022). Pyroptosis is inflammatory cell death form that results in inflammatory cytokine massive release and cell rupture. Recently, pyroptosis reveals a significant role in LPS-induced ALI and excessive inflammation (Liu et al., 2022, Zhang et al., 2022). Pyroptosis results from the inflammatory caspases release, pore-forming gasdermins, and activating inflammasomes, particularly NLRP3. The NLRP3 inflammasome activation induces the release of active Casp-1 resulting in the cleavage of active GSDMD-N (gasdermin D) to execute pyroptosis (Kang et al., 2022). Consistently, our data indicated the enhancement of NLRP3/Casp-1/IL-1β signaling after LPS injection. This was accompanied by an increase in GSDMD-N indicating pyroptosis. These effects were inhibited in KSG pretreated groups. Collectively, these data suggest that KSG exerts its anti-inflammatory effect mainly via prohibiting NLRP3/NF-κB signaling axis. The anti-inflammation capacity of KSG is similar to that of other kaempferol 3-O-β-sophoroside derivatives that has been shown in human endothelial cells (Kim et al., 2012, He et al., 2022).

Previous studies clarified the critical role of oxidative stress in ALI as disturbance of redox balance eventually leads to biochemical and metabolic intracellular dysfunction. Administration of LPS induces the excessive generation of ROS which can directly damage different organelles of the cell, inducing lipid peroxidation as well as promoting the pro-inflammatory response accentuated by oxidative stress (El-Agamy, 2011, Shaaban et al., 2018). Nrf2/HO-1/NQO1/GCLc signaling is one of the most crucial endogenous antioxidative stress pathway that significantly controls the severity of oxidative and inflammatory response in inflammation-related disorders (Kang et al., 2022). Normally, Nrf2 is present in cytosol at low cellular concentrations as it is negatively captured by Keap1. Once the cell is exposed to oxidative damage, Nrf2 is activated, liberated, and moved into the nucleus to activate downstream antioxidant genes as HO-1, NQO-1 and GCL. To date, compelling evidence indicates the regulatory role of Nrf2 signaling and associated oxidative inflammatory response in the LPS-caused ALI pathogenesis (Huang et al., 2020, Luo et al., 2022).

In line with former-mentioned studies, our results demonstrated that LPS constrained the activity and expression of Nrf2/HO-1 which impacted the cellular oxidative state. The suppression of Nrf2 signaling was concomitant with increase the lipid peroxidative products (MDA/PC/4-HNE), and impairment in the anti-oxidative capacity (CAT/GSH/SOD) in the lung tissues during LPS-induced ALI. Contrarily, KSG pretreatment enhanced mRNA expression of Nrf2/HO-1/NQO1/GCLc and Nrf2 protein expression as well as its binding activity. These data clarified the potent activity of KSG in restraining septic ALI via activation of Nrf2-dependent anti-oxidative machinery.

In computational studies, our investigation aimed to elucidate the interactions between KSG and the KEAP1 protein, shedding light on potential mechanisms for inhibiting Keap1-Nrf2 protein–protein interactions. Through the utilization of molecular docking, the accuracy of the approach was assessed by comparing the Root Mean Square Deviation (RMSD) values of the co-crystallized and re-docked ligands, resulting in an RMSD value of 0.7199 Å, indicating the precision of the docking methodology. Subsequently, the examination of docking scores and MM-GBSA binding free energies emphasized the superior performance of KSG over the reference inhibitor, showcasing its enhanced binding affinity and thermodynamic stability. Similar patterns, including hydrogen bonds, water bridge interactions, and hydrophobic contacts, within the KEAP1 binding site were revealed through molecular interaction analyses, providing valuable insights into the specificity and strength of the ligand-receptor interactions.

Moving to MD studies, dynamic interactions of docked complexes over a 100-nanosecond simulation period were explored. The stability of KSG and the co-crystal ligand with KEAP1 was demonstrated by Root Mean Square Deviation (RMSD) analyses, indicating that the complexes consistently maintained RMSD values below 1.4 Å. This stability persisted throughout the simulation, as evidenced by Root Mean Square Fluctuation (RMSF) analyses, which indicated minimal structural variability for both KSG and the co-crystal ligand. Further insights into molecular interactions during MD simulations revealed dynamic behaviors, with significant contributions to ligand stability and interaction patterns demonstrated by specific residues like TYR334, ASN382, and ARG415. These findings collectively provide a comprehensive understanding of the dynamic and static aspects of KSG and the reference inhibitor within the KEAP1 binding site, informing potential implications for modulating Keap1-Nrf2 interactions.

However, this study investigated only the protective activity of KSG, and could not clarify the ability of KSG to reverse LPS-induced ALI if KSG is administered after LPS challenge. In addition, the specific role of KSG against apoptotic pathway should be investigated. Finally, the long-term use of KSG, its adverse effects as well as its impact on patients are also worthy of further investigation**.**

## 5 Conclusion

Collectively, our study demonstrated the protective capacity of KSG against ALI induced by LPS in mice. KSG possesses potent anti-inflammatory activities that modulate the NF-κB/NLRP3 signaling and hence suppressed LPS-associated inflammatory response. Additionally, KSG enhanced Nrf2/downstream antioxidant genes and hence conferred protection against oxidative damage which may in-part participate in further restraining inflammatory response. Our comprehensive computational investigation establishes KSG as a promising disruptor of Keap1-Nrf2 interactions, shedding light on its potential as a therapeutic agent in modulating cellular responses through the Nrf2 pathway. KSG showed favorable binding characteristics and sustained stability observed in the *in-silico* simulations. The present data encourages further investigation of the potential pharmacological activities of KSG as it might be a new candidate for inflammatory disorders.

**Funding**

The Deanship of Scientific Research (DSR) at King Abdulaziz University (KAU), Jeddah, Saudi Arabia has funded this project, under grant no. (RG-100–130-43).

**Ethical approval**

The study protocol was approved by the Research Ethical Committee of 10.13039/501100024252Faculty of Pharmacy, 10.13039/501100004054King Abdulaziz University under the number PH-116-40.

## CRediT authorship contribution statement

**Wesam H. Abdulaal:** Writing – review & editing, Supervision, Resources, Project administration, Investigation, Funding acquisition, Formal analysis, Data curation. **Ulfat M. Omar:** Writing – review & editing, Supervision, Resources, Project administration, Funding acquisition, Formal analysis, Data curation. **Mustafa Zeyadi:** Writing – review & editing, Supervision, Resources, Project administration, Investigation, Funding acquisition, Formal analysis. **Dina S. El-Agamy:** Writing – review & editing, Writing – original draft, Validation, Methodology, Investigation, Formal analysis, Data curation, Conceptualization. **Nabil A. Alhakamy:** Writing – review & editing, Supervision, Resources, Project administration, Investigation, Funding acquisition, Formal analysis, Data curation. **Naif A. R. Almalki:** Writing – review & editing, Formal analysis, Data curation. **Hani Z. Asfour:** Writing – review & editing, Supervision, Formal analysis, Data curation. **Mohammed W. Al-Rabia:** Writing – review & editing, Supervision, Formal analysis, Data curation. **Abdulrahim A. Alzain:** Writing – review & editing, Writing – original draft, Software, Methodology, Formal analysis, Data curation, Conceptualization. **Gamal A. Mohamed:** Writing – review & editing, Software, Formal analysis, Data curation. **Sabrin R.M. Ibrahim:** Writing – review & editing, Writing – original draft, Software, Formal analysis, Data curation.

## Declaration of competing interest

The authors declare that they have no known competing financial interests or personal relationships that could have appeared to influence the work reported in this paper.
