# Supplementary material for: Protective effect of kaempferol glucoside against lipopolysaccharide-caused acute lung injury via targeting Nrf2/NF-κB/NLRP3/GSDMD: Integrating experimental and computational studies
Source: Saudi Pharm J. 2024 Apr 18;32(6):102073. doi: 10.1016/j.jsps.2024.102073 (PMC11046126; doi:10.1016/j.jsps.2024.102073)
Supplement: Supplementary Data 1 [file mmc1.docx]

**Supporting Materials**

Protective effect of kaempferol glucoside against lipopolysaccharide-caused acute lung injury via targeting Nrf2/NF-κB/NLRP3/GSDMD: Integrating experimental and computational studies

**Table S1.** Primer sequences used in RT-PCR measurements.

| Gene  (Mouse) | Accession # | Sense primer (5ʹ-3ʹ) | Antisense primer (5ʹ-3ʹ) | PCR Product (bp) |
| --- | --- | --- | --- | --- |
| *TNF-α* | AY423855 | TCCCAAATGGCCTCCCTCTC | TACGACGTGGGCTACAGGCT | 99 |
| *NLRP3* | NM_145827 | TGGGTTCTGGTCAGACACGAG | GGCGGGTAATCTTCCAAATGC | 299 |
| *Casp-1* | NM_009807 | GGACCCTCAAGTTTTGCCCT | GCAAGACGTGTACGAGTGGT | 103 |
| *IL-1β* | NM_008361 | TGCCACCTTTTGACAGTGATG | TGATGTGCTGCTGCGAGATT | 138 |
| *Nrf2* | [NM_010902](http://www.ncbi.nlm.nih.gov/nuccore/NM_010902) | AAGAATAAAGTCGCCGCCCA | AGATACAAGGTGCTGAGCCG | 170 |
| *HO-1* | [NM_010442](http://www.ncbi.nlm.nih.gov/nuccore/NM_010442) | GAAATCATCCCTTGCACGCC | CCTGAGAGGTCACCCAGGTA | 122 |
| *NQO1* | [NM_008706](http://www.ncbi.nlm.nih.gov/nuccore/NM_008706) | CATTGCAGTGGTTTGGGGTG | TCTGGAAAGGACCGTTGTCG | 111 |
| *GCLc* | [NM_010295](http://www.ncbi.nlm.nih.gov/nuccore/NM_010295) | CTTTGGGTCGCAAGTAGGAAGC | GGGCGTCCCGTCCGTTC | 182 |
| *β-actin* | [NM_007393](https://www.ncbi.nlm.nih.gov/entrez/viewer.fcgi?db=nucleotide&id=930945786) | ACTGTCGAGTCGCGTCCA | TCATCCATGGCGAACTGGTG | 88 |
